# Exploration of knowledge and understanding in patients with primary adrenal insufficiency: a mixed methods study

**DOI:** 10.1186/s12902-017-0196-0

**Published:** 2017-08-01

**Authors:** L. M Shepherd, A. A Tahrani, C Inman, W Arlt, D. M Carrick-Sen

**Affiliations:** 1 Department of Diabetes & Endocrinology, Heart of England NHS Foundation Trust, Birmingham Heartlands Hospital, Bordesley Green East, Birmingham, West Midlands B9 5SS UK; 2 Institute of Metabolism & Systems Research, University of Birmingham, IBR Tower, Level 2, Edgbaston, Birmingham, West Midlands B15 2TT UK; 3 Department of Public Health & Community Health, Birmingham City University Faculty of Health, City South Campus, Westbourne Road, Birmingham, B15 3TN UK; 4 Birmingham Health Partners, University of Birmingham & Centre for Endocrinology, Diabetes and Metabolism (CEDAM), Birmingham, B15 2TT UK; 5 School of Nursing, Institute of Clinical Sciences, College of Medical & Dental Sciences, University of Birmingham, Edgbaston, Birmingham, West Midlands B15 2TT UK; 6 Heart of England NHS Foundation Trust, Birmingham Heartlands Hospital, Bordesley Green East, Birmingham, B9 5SS UK

**Keywords:** Addison’s disease, Adrenal insufficiency, Nursing, Knowledge, Adherence, Hydrocortisone, Acute illness, Stressful event

## Abstract

**Background:**

Primary adrenal insufficiency (PAI) is a rare and severe condition requiring lifelong steroid replacement. During acute illness or stressful events, it is important to appropriately adjust glucocorticoid dose; failure to do so may lead to an adrenal crisis. The aim of the study was to explore patients PAI knowledge and understanding of the condition, steroid replacement adjustment during acute illness or stress and provided education.

**Methods:**

Ten adult patients with PAI were purposefully recruited from two hospitals in a tertiary NHS Trust in England, UK. Data was collected using a mixed method approach utilising semi-structured audio-recorded interviews and hospital case note review. Interviews were transcribed verbatim and analysed using Burnard’s content analysis framework. Information from the hospital case note review was captured using a matrix table based on pre-defined criteria.

**Results:**

Four key themes emerged: ‘Addison’s disease and hydrocortisone replacement’; ‘stress and corticosteroids’; ‘patient compliance/adherence’ and ‘transition’. Patients reported feelings of ‘going through a transition from uncertainty to adaption’ following diagnosis. All participants had a good level of knowledge and understanding of required medication however application in times of need was poor. Medication adherence and prevention of a crisis relied not only on patient knowledge and application but also the support of family and health professionals. Health care professional knowledge required improvement to aid diagnosis and management of PAI.

**Conclusion:**

Patients with PAI did not apply existing knowledge to adjust steroid dose during acute illness or stress. Although a sample of limited size, our study identified there is a need to further explore why patients with Addison’s disease do not apply existing knowledge during times of increased need. Future research should consider appropriate behaviour change interventions to promote medication adherence to reduce risk of an adrenal crisis.

## Background

Primary adrenal insufficiency (PAI) or Addison’s disease is a life threatening endocrine condition, where the destruction of the adrenal cortex, most commonly via an autoimmune mechanism results in inadequate production of glucocorticoids, mineralocorticoids and androgens [1]. The prevalence of PAI is 10–22 per 100, 000 [2, 3] and an incidence of 4–6 per million per year [4, 5].

In times of acute illness, for example flu like illness and fever, patients need to increase their glucocorticoid dose to mimic physiological changes in cortisol secretion during stress [6, 7]. The occurrence of adrenal crisis in PAI has been reported as 5.2–8.3 crisis/100 patient years [6–8]. About 55% of women and 52% of men reported one or more adrenal crises since diagnosis [7]. Whilst those who have experienced a previous adrenal crisis are at greater risk of a subsequent episode [6, 7].

PAI is a chronic condition requiring adherence and appropriate adjustment of medication in times of need to prevent an adrenal crisis and hospital admission [6, 7]. Adherence and adjustment of medication is linked to patients knowledge and understanding. Within other chronic conditions like diabetes, adequate knowledge and understanding has been shown to increase medication adherence [9]. In PAI, health professional and patient guidance is available regarding the adjustment of steroids during acute illness and stress [2, 10]. However, there is limited available evidence of level, comprehension and application of knowledge in patients with PAI. Furthermore, it has been identified that adrenal crisis still occurs even in patients with chronic adrenal insufficiency who have been educated [6].

Hence, the aims of our study were to explore if patients with PAI have sufficient knowledge and understanding of the condition; knowledge of how and when to adjust steroid replacement during acute illness or stressful event; and been provided with the required information**.**

## Methods

A mixed method study was conducted involving qualitative semi-structured interviews and review of participant’s health care records. Qualitative researchers utilising interviews seek to understand rather than explain [11] and focus on the illumination of data through the subjects interpretation of their circumstance.

### Sample/participants

Pragmatic purposive sampling was adopted from two different demographic location hospitals in a single tertiary NHS Trust in UK. It was planned to recruit up to 15 participants, or until data saturation was achieved [12, 13]. It has been posited by Guest et al. (2006) that data saturation occurs by the time 12 interviews have been analysed [13]. Therefore the planned recruitment number allowed for attrition and allowed for further data to be gathered until data saturation was achieved. However, data saturation occurred at the point when ten patients had participated in the study.

Patients were approached in the waiting area of the endocrine clinic when attending their appointment by a health professional/receptionist who was not involved with the research and were provided with a patient information sheet. They contacted the researcher at a later date if they were interested in participating. Inclusion criteria included; participants 18 years old or over, English speaking and with an established diagnosis of PAI based on endocrinologist diagnosis, and usually administers their own medication. The leading author is an endocrine specialist nurse at the centre and hence she was known to the patients.

### Data collection

Individual face-to-face, semi structured interviews and review of participant’s health care records were carried out by the first author in this study. Prior to commencement of the interview discussion took place of the purpose of the study, participant involvement and consent was attained. The right to withdraw at any time was reiterated. Interviews lasted up to 60 min and were conducted at a location of the participant’s choice. A conversational interview technique was utilised with the aid of a semi structured interview guide (Table 1) as recommended by Holloway & Wheeler [14]. An interview guide was developed from key themes in the literature and from the clinical researchers’ experience in the field. This interview guide allowed flexibility to accommodate new topics and concepts introduced by the participants [14].

**Table 1:** The interview guide

The following questions were used as an interview guide. Commencing with an icebreaker, questions may be asked in a different order, omitted or added depending on participants’ responses.
1. How long have you had Addison’s disease? Tell me more. Diagnosed when and how? Medication?
2. Have you been advised what to do with your medication in times of stress/illness? Who gave the advice? When? Give me examples what you would do when you experience
-a cold
-a temperature
-an infection
-diarrhoea
-vomiting
-psychological distress, e.g. car accident without injury, bereavement
-surgery
-dental treatment
3. Have you ever been advised to stop your steroids? By whom? When?
4. Have you ever been advised to carry or wear anything stating your illness/medication?
5. Have you ever been in contact with a support group? What information/advice did they give you?
6. Do you have an emergency hydrocortisone injection kit? Have you ever needed to use this? If not, is this something you would consider having?
7. Have you ever had an Addisonian crisis? Tell me what happened? What precipitated this? What happened when you got to hospital?
8. What education/advice would you like to have had when diagnosed? On a regular basis?
9. Do you have to take anything into account about your illness if you are going on holiday?
10. Is there anything else you would like to talk about/add?

All participant health care records were retrospectively reviewed to assess if there was documented evidence of education provided from endocrinologists and endocrine specialist nurses including; sick day rules, provision of steroid card, wearing of medic alert jewellery and possession of emergency hydrocortisone injection kit (Table 2).

**Table 2:** Documented evidence of education

Participant number	Documentation of sick day rules	Steroid card	Medical identification jewellery	Emergency hydrocortisone injection kit
1	Y	Y	Y	N
2	Y	N	Y	N
3	Y	Y	N	N
4	Y	Y	N	Y
5	Y	Y	Y	Y
6	Y	Y	Y	N
7	Y	Y	Y	Y
8	N	N	N	N
9	N	N	N	N
10	Y	Y	N	N

‘Y’ = Yes patient was given information; ‘N’ = no information was not provided to patient

### Data analysis

Guidance on the data analysis was based on Burnard’s [15, 16] thematic content analysis framework. This involved a stepped process of identifying themes and categories that ‘emerge from the data’ [16]. Analysis of the data involved utilising steps one to ten.

The structure of Burnard’s data analysis led the reduced final categories, sub categories shown in (Table 3) with corresponding examples of verbatim transcript. During data analysis it became evident that no new categories or sub categories were being identified and that data saturation had been achieved, and subsequently recruitment concluded at 10 participants [13].

**Table 3:** Final categories and subcategories

Categories	Subcategory
Addison’s Disease & Replacement	Signs & symptoms
	Crises
	Unwanted effect of medication
	Associated conditions
Stress & Corticosteroids	Adjustment of medication
	Emergency injection kit
	Emergency identification
	Stopping steroids
Patient adherence	Education
	Support systems-
	Family
	Professional
	Voluntary
Transition	Reeling
	Dealing
	Healing

### Rigour of data collection

In order to improve rigour the principles of trustworthiness as described by Lincoln & Guba, Holloway & Wheeler and Polit & Beck were adopted [14, 17, 18]. Dependability of the interviews was enhanced by the use of an interview guide that was developed based on relevant literature. With regard to credibility, the first author reconfirmed the content of the interview with the participant to determine accuracy of understanding and allowed them to add further data to complement the researchers understanding, along with the utilisation of participants verbatim quotes [14]. Field notes were maintained regarding setting and participant. An auditable decision trail was maintained by documenting raw data as well as sources of data generation and analysis decisions [14]. Data gathered was peer reviewed by academic supervisors at various stages of the research process allowing for verification of the effectiveness of the data collection procedure, comprehensiveness of descriptions, inclusivity of samples and logic of arguments [19].

## Results

Two male and eight female participated in the study with a mean age of 47 years (age range 21 to 63 years) and a median duration of PAI of 19 years (range 3–46 years). All were White Europeans. Seven participants had previous hospital admissions due to an adrenal crisis. Most participants described their journey from diagnosis to present.

The results presented below leads with each of the four categories as identified in (Table 3). Each one is introduced, supported by verbatim quotes from participants and accompanied by a commentary. The discussion with previous literature follows the results section.

### Category 1: Addison’s disease and hydrocortisone replacement

Participants identified **signs and symptoms** of PAI prior to diagnosis. This was predominately due to the severity of symptoms experienced prior to diagnosis and/or adrenal crisis. All of the respondents described the length of time to diagnosis as a problem, receiving many differential diagnoses, despite seeking medical attention on several occasions prior to diagnosis. This is demonstrated in the below verbatim quotes,

“…I used to go the football…and half way through the first half I had to sit down. If I used to go the pub I used to have to sit down…I was too tired to stand up.” (P5).

“I just kept looking at them thinking why are my hands brown. Why are these brown on me?” (P6).

“The Doctor did a glandular fever test which was clear and then he decided I was depressed.” (P1).

“…they kept saying it was the effect of septrin or was I pregnant or had I got anorexia (laughter) and all sorts of different things…” (P7).

Seven out of ten participants affirmed they had experienced an adrenal **crisis** since diagnosis and required intravenous or intramuscular hydrocortisone and/or hospitalisation.

“…I was getting to the stage I couldn’t talk to … because my body was shutting down 'cause I was that bad…” (P1).

“…I was on the stretcher and they couldn’t get my blood pressure in the ambulance…” (P3).

Participants described the **unwanted effect of medication**. Whilst participants were aware of potential side effects of their medication they only spoke about the effect steroids had on bone health.

“…osteoporosis which is obviously caused by the steroids cause I’ve read up on that…” (P3).

Only three participants discussed **associated conditions** of Addison’s disease with other autoimmune conditions despite all participants having other autoimmune conditions.

“…you’re thinking because it’s in the family of diabetes, isn’t it you know? When you’ve got thyroid and then diabetes and that, you’d think they’d catch it easy…” (P6).

“…I didn’t know about the thyroid when I first had it and diabetes and some of the other things.” (P7).

### Category 2: Stress & corticosteroids

Participants discussed their understanding of the need to **adjust medication**, which overall was very good. Nine out of the 10 patients knew how to adjust their medication in times of intercurrent illness. Medication intake was bound to a prescribed rigid treatment regimen which required rather complex and demanding self-care. However, length of time since diagnosis was not related to increased knowledge as demonstrated in the verbatim quote from participant 8.

“No, no, not at all. Funnily enough I did not realise you could, you should or you needed to, to be honest” (P8).

“…the only thing I've ever been told is if you’re abroad on holiday and you have stomach problems you know, if you have diarrhoea or something like that, then take an additional tablet but that’s as much as I've been told…”(P8).

“I’ll double them and then if I have a temperature or anything else double it until the infections gone.” (P4).

Respondents informed HCPs of their condition, and also relied upon them for advice and extra medication during dental and surgical procedures. They saw the HCP as the ‘expert’ and were guided by their recommendation, even if they knew this to be incorrect.

“Yes I did have to tell them…when they knew I got Addison’s disease they said that I would have to go over night ‘cause I would have to have steroids before I went into surgery. I’d have to stay in that extra bit longer probably just as extra day to boost me up…” (P9).

“I think it depends on which Doctor you see as well. Like when I’ve been to clinic and I like to see one Doctor now rather than each time I come to clinic to see a different Doctor and they tell you different things and I don’t think they know that much about it…”(P7).

Two participants currently had an **emergency hydrocortisone injection kit** but six expressed an interest in having one.

“Major problems, yes, I would certainly use it if I was taught how to use it…” (P8).

“I did have one when I was with the old doctor.” (P7).

Regarding **emergency identification** reassuringly all study participants carried a steroid card and/or wore medical information jewellery.

“Yes, when I was first diagnosed they wouldn’t let me out of the hospital unless I got a bracelet.” (P7).

“I wear a meditag saying what’s wrong with me and that I’m on steroids.” (P10).

Worryingly two participants had been advised to **stop taking their steroids** either by a healthcare professional or friend at some point since their diagnosis.

“…I couldn’t hold anything down, she [G.P] said don’t worry it won’t hurt you to miss them for once cause you’ve got bits of steroid going round your body… but she shouldn’t have told me that.” (P6).

“…not medical people but other people said why don’t you go to a homeopath. At the time I didn’t realise…” (P10).

### Category 3: Patient adherence

There was a noticeable difference in the quality and quantity of **education** provided to participants on pre and post diagnosis from healthcare professionals. Several respondents felt they were given insufficient knowledge and advice and indicated that they would have liked to have received more information. None stated they received an adequate amount.

“…when I got diagnosed I felt a bit as though I wasn't told much about it…I had to go and find it myself…I kept having to ask people.” (P6).

“…the consultant that diagnosed me at …said this condition would have to be managed throughout my life…” (P4).

Three **support systems; family, healthcare professionals and voluntary** were identified as important to, and relied upon by, the participants. **Family** provided substantial support when it came to the management of the participants’ condition and also when seeking a diagnosis for the condition. The diagnosis was often only established after perseverance from family in seeking medical help. Whilst two participants felt their family should have a better understanding of the condition, most participants relied upon the opinion and assertiveness of family during intercurrent illnesses. This was related to the administration of emergency treatment when seeking urgent medical attention.”

“[At the hospital] … my husband said look I don’t want it to go that far now, I want you to deal with it now.” (P9).

“…if it weren’t for my Mom really I was just going to lie here and die.” (P2).

Participants felt that rapport with the **healthcare professional** contributed a major role in the support received. However other participants felt they did not always receive professional support.

“… he said I’ve never ever diagnosed or treated anybody with Addison’s disease…” (P6).

“…it’s a lot easier have someone sit there you know. It’s a bit strange isn’t it when you see a different Doctor…” (P1).

Support from **voluntary** self-help groups was not always seen as a priority by participants with only one being a member, although some had accessed information through the World Wide Web.

“I’ve looked on the internet though…yes and I’ve printed off the first thousand pages (laughter)” (P5).

“I look it up on the internet; I’ve looked it up on there.” (P7).

### Category 4: Transition

Participants described a period of transition including the psychological progression involved in adapting to change, from pre diagnosis to their present situation going through a process of ‘reeling’, ‘dealing’ and ‘healing’.

Participants described the first stage as ‘**reeling**.’ Chronic illness may cause a finale to a recognizable existence represented by the time at which the diagnosis of PAI was made. At this point participants tried to establish ways of dealing with their condition, in order for the diagnosis to be incorporated into their lives. Participants felt disbelief and shock.-

“…when I was first diagnosed I did feel really, really depressed.Why has this happened to me?” (P2).

“…when I was diagnosed it was a big shock ‘cause it was going to affect the rest of my life…” (P4).

Participants experienced **‘dealing’** as a static stage and during which time they reported feeling overwhelmed at being different from others who did not have the disorder. Respondents stated they felt isolated and alone, thinking no one understood their experience and found difficulty in explaining how they felt. It was a time of self-absorption as the task was to try and reclaim themselves in the midst of change and to deal with the diagnosis and illness. Some chose to ignore their diagnosis and others rejected offers of assistance during this time. What became evident in this theme was the isolation felt following diagnosis where participants sought to find answers:

“…I remember thinking I wish I knew somebody else who had it so they could tell me I’m going to be alright and I can lead a relatively normal life…” (P9).

“…I used to write about six questions down and I’d go in and ask these questions…” (P3).

Participants experience a **healing** stage when they felt they had become ordinary again, even though the condition remained. Here the participants were more open to learning from life and took action on issues that confronted them. Therefore this stage demonstrated participants acceptance of the diagnosis and adaptation:

“…I say it’s the one thing in my life that I’m comfortable with … You’ve just got to live with it and that’s it really.” (P8).

“No it’s all pretty much get on normally really like it’s I still cannot believe how two little tablets can make a difference to the way you feel.” (P5).

### Health care record review

Data was collected from participant’s health care records. This included all available past and current healthcare records which encompassed information from all healthcare consultations and typed correspondence to and from the general practitioner. Quality of documentation varied and was in the main legible but brief but overall inadequate. Table 2 illustrates the data collected from documented evidence of education from the health care record review. The education was delivered by a combination of endocrinologists and endocrine specialist nurses.

It was documented that eight participants had received advice on ‘sick day rules’ during times of stress. Interestingly, participant eight had said they received very minimal medication adjustment advice and this was not documented in their health care records. Participant nine also had no documented evidence of ‘sick day rule’ advice, but reassuringly, demonstrated a good understanding of dose adjustment.

It was recorded that seven patients had steroid cards, with no documentation stating that patient two, eight or nine had been advised to carry one. However, encouragingly all participants were in possession of one.

Only six participants wore medical identification jewellery and five had documentation that they had been advised to wear it.

There was documented evidence that three participants were in possession of an emergency hydrocortisone kit, although respondent seven no longer did so. However, there was no evidence that the participants had received any education on the preparation and administration of this, or if they were deemed competent to administer when required.

It is worth noting that it was the two participants that had been diagnosed the longest duration, had least documentation in the notes.

Overall, documentation of sick day rules and possession of steroid cards was good.

## Discussion

The study revealed that a majority of the interviewed patients with PAI at this tertiary UK centre have good knowledge and understanding of the condition. Although the generalizability of the study is limited due to the small sample size, the study demonstrated the application of knowledge with regards to medication adjustment in times of need is limited and concerning. Further, participants describe their experience as a transitional journey from pre diagnosis to present, through three phases; reeling, dealing and healing. Information obtained from health care records was variable regarding confirmation of patient education that had been provided and was known by the patient. Finally, there also appears to be inconsistency in terms of health professional knowledge, and advice given.

### Knowledge

The present study showed that it is not only patients with PAI who require knowledge of the condition, replacement therapy, and importantly the adjustment of glucocorticoids during intercurrent illness, but also the support mechanisms the participants rely upon. These include family, HCPs and voluntary.

Our study findings demonstrate that HCPs are seen as experts in the knowledge and management of health conditions by patients and family, and therefore it is important that HCP’s knowledge is adequate and up to date to diagnose and manage related life threatening events, and complications [7].

### Health professional knowledge in rare conditions

Findings from the present study confirm that participants received many differential diagnoses prior to the establishment of the diagnosis of PAI. This resulted in most patients having a treatment delay, which comes with an increased risk of adrenal crisis, and this finding is confirmed in the literature [20]. Educating health care professionals is essential [21, 22].

The very fact that these diseases are rare means that HCPs are unlikely to have encountered patients with the condition. This is particularly true within the primary care setting [23], where patients ultimately first present with signs and symptoms of PAI. It is therefore not just patients who need access to high quality information, and demonstrates the importance of the HCP knowledge in the diagnosis, management and continual support that patients with rare diseases require [5, 24, 25].

It is acknowledged that only those living with a rare condition will have the direct experience of how it affects them and their family [25]. Subsequently, the patient is seen as the expert. However, our study findings demonstrate that patients look to HCPs for on-going support in their journey, and shared decision making between patients and HCPs is fundamental in healthcare and aids both parties. It requires the medical problem to be identified and reasonable options laid out, primarily by the physician [26]. Although patients are responsible to identify and convey their goals and concerns relevant to the decision they are facing. In summary, patients and healthcare professionals have an important role and must be receptive to each other’s input.

Our findings demonstrated that during the healing stage participants had accepted the diagnosis and had adapted their lives to feel ‘ordinary again’. There is strong evidence that engaged patients are more informed and likely to fully consider risk and benefit of different treatment options [26], although there is also evidence that not all patients want to be involved in healthcare decisions [27]. Therefore it is important to consider that some patients will still rely on HCPs for the guidance and support of the condition. Ultimately, participants are responsible on a day-day basis for the management of the condition.

### Patient knowledge and application

Our study confirmed that patients have a good knowledge base of how to adjust glucocorticoid treatment during acute illness as shown by their responses to question 2 of the interview schedule (Table 1), which contrasts a number of studies that have highlighted knowledge deficits of adrenal insufficiency, crisis, management and prevention [7, 28, 29]. However, what our findings do demonstrate is that despite good knowledge, participants still experienced adrenal crisis following diagnosis. This suggests that knowledge does not translate into behaviour change, resulting in a gap in knowledge-application. This is in keeping with findings by Hahner et al. [6] and Van der Meij et al. [30] who found education sessions do not achieve sufficient self-management of glucocorticoid replacement. It is important therefore to explore why educated patients with PAI do not apply information provided in times of need.

### Documentation of the education provided to patients

This study showed that documentation in the medical notes regarding the education provided to patients with PAI was good but detail inadequate. That could be due to the lack of an agreeable standardised format of recording the different components of the education given to the patient. However, the lack of recording may not necessarily reflect lack of education as in our study 9/10 patients knew what to do in acute illness despite the variable recording of the education information in the medical records. Patients may receive education and subsequently attain knowledge from a variety of other sources other than the healthcare professional, for example, the World Wide Web, self-help groups, family and friends. This is supported by Sorensen et al. [31], who found that errors in the data may reflect incorrect data entry or lack of entry of available information. Also while the original source of information may be correctly entered into the data source it may not reflect the true condition or characteristic of the subject [31]. This calls for improvement in the standardisation of documentation of patient education given.

### Experience - the journey

The present study uniquely describes the patient’s experience of a rare condition, PAI. They describe the transitional journey within the context of ‘reeling’, ‘dealing’ and ‘healing.’ These are similar concepts to those described by Kubler-Ross [32] within the grief process which include denial, anger, bargaining, depression and finally, acceptance. Coping with a new diagnosis involves implementation of strategies that enable one to assess, reflect and adapt. Isla Pera et al. [33] report similar findings in a study of people with type 1 diabetes mellitus. The study describes patient and relatives experience as an emotional reaction similar to that of the grief cycle.

The present study describes the initial disbelief and shock participants feel when receiving the diagnosis. They begin to establish ways of dealing with the diagnosis and treatment regimens and incorporate this into their lives. Forss et al. (2012) also found that glucocorticoid replacement regimens impacted on patients’ lives and their self-perceived outcomes, such as physical activity or family life [34]. It is recommended HCPs talk about disease adaptation rather than acceptance, since patients need to reconstruct their identity to the situation [33]. Within Isla Pera et al. [33] study, health professionals related patient poor adherence to denial of the disease, conversely patients reported poor adherence related to the demands of treatment and its impact on quality of life. However, there is a link between reassurance and adaptation, and participants of the present study valued and required on-going support also highlighted by Boot et al. [35]. However, acceptation of a disease and its limitations are a necessary step in the adaptation process, and worried patients need reassurance in order to achieve adaptation [35]. In the present study it was interesting to note that patients reported receiving reassurance from family rather than healthcare professionals when dealing with the condition day to day and in times of crisis.

The present study participants felt overwhelmed and isolated during the ‘dealing’ stage and sought to find answers. Failure from healthcare professionals to react appropriately, led to patients feeling isolated. Participants either wore or carried something identifying they were taking steroids. Wearing of medical identification jewellery is recommended [2, 10], and provides emergency HCPs responsible for the urgent care of a patient with PAI, with critical information that will guide additional assessments and medical interventions. The prevalence of patients with chronic disorders who use medic alert jewellery is unknown [36] and available data is paediatric focused [36, 37]. However, patients’ view of medical alert jewellery varies [37, 38]. Some see themselves as ‘belonging’ to a particular special group and therefore view it as positive, for others it is negative, being viewed as ‘different’ from others, and labelled by a diagnosis, which can lead to isolation [38].

Turner & Kelly [39] state that the emotional dimensions of chronic conditions are often overlooked when medical care is considered. Since PAI is a chronic condition, HCPs have a fundamental role in guiding patients and their family through this transitional process, recognising the multiple psycho-social dimensions of chronic disease in order to address both their physical and emotional needs. It is important to remember that the relationship between the HCP, patient and family should be a continuum and not time specific.

### Limitations

The research was conducted in one NHS Trust and therefore findings may not be generalisable to other healthcare provider organisations. The sample size was small, and does not represent the gender distribution of PAI; larger samples are required to validate findings. The impact of gender and age on our findings would be an interesting area to explore in future studies as our current sample size does not allow us sub-group analysis. Future research should also explore the impact of age and disease duration and its effect on education and prevention of adrenal crisis. It is not known how many healthcare providers educated the patients over the course of the patients care.

The lead researcher who also was the interviewer was known to participants and therefore it is important to consider this relationship and its potential impact. There are numerous kinds of relationships that might enter into qualitative research; therefore, it is essential not to ‘hide behind the mask of rapport’ or the ‘wall of professional distancing’ [40]. As qualitative researchers we must be trustworthy, transparent and reflexive in communications with participants and “honour the consequence of acting with genuineness” [40] (p. 105).

## Conclusion

This is the first study where patients have described their experience of PAI as a transitional journey. Although the study findings are not generalizable, it highlights factors that influence the diagnosis and management of the condition. We found that provision and receipt of patient knowledge does not necessarily result in a behaviour change to apply the knowledge in times of need; the reasons for which are currently unknown. This lack of application of knowledge is likely to be multifactorial including patient related factors and possibly problems with interactions with healthcare professionals. Future studies should explore the reasons underpinning this lack of application in knowledge in order to develop appropriate interventions.

## Abbreviations

HCP: Health Care Professional.
PAI: Primary Adrenal Insufficiency
